# Dietary Plants for the Prevention and Management of Kidney Stones: Preclinical and Clinical Evidence and Molecular Mechanisms

**DOI:** 10.3390/ijms19030765

**Published:** 2018-03-07

**Authors:** Mina Cheraghi Nirumand, Marziyeh Hajialyani, Roja Rahimi, Mohammad Hosein Farzaei, Stéphane Zingue, Seyed Mohammad Nabavi, Anupam Bishayee

**Affiliations:** 1 Office of Persian Medicine, Ministry of Health and Medical Education, Tehran 1467664961, Iran; mina_nirumand@yahoo.com; 2 Pharmaceutical Sciences Research Center, Kermanshah University of Medical Sciences, Kermanshah 6734667149, Iran; marziyeh.alyani@gmail.com; 3 Department of Traditional Pharmacy, School of Traditional Medicine, Tehran University of Medical Sciences, Tehran 1416663361, Iran; rojarahimi@gmail.com; 4 Department of Life and Earth Sciences, Higher Teachers’ Training College, University of Maroua, Maroua 55, Cameroon; stephanezingue@gmail.com; 5 Department of Animal Biology and Physiology, Faculty of Science, University of Yaoundé 1, Yaounde 812, Cameroon; 6 Applied Biotechnology Research Center, Baqiyatallah University of Medical Sciences, Tehran 1435916471, Iran; nabavi208@gmail.com; 7 Department of Pharmaceutical Sciences, College of Pharmacy, Larkin University, Miami, FL 33169, USA

**Keywords:** nephrolithiasis, dietary plants, urolithiasis, natural dietary supplement, phytochemicals, kidney stone

## Abstract

Kidney stones are one of the oldest known and common diseases in the urinary tract system. Various human studies have suggested that diets with a higher intake of vegetables and fruits play a role in the prevention of kidney stones. In this review, we have provided an overview of these dietary plants, their main chemical constituents, and their possible mechanisms of action. *Camellia sinensis* (green tea), *Rubus idaeus* (raspberry), *Rubia cordifolia* (common madder), *Petroselinum crispum* (parsley), *Punica granatum* (pomegranate), *Pistacia lentiscus* (mastic), *Solanum xanthocarpum* (yellow-fruit nightshade), *Urtica dioica* (stinging nettle), *Dolichos biflorus* (*horse gram*), *Ammi visnaga* (khella), *Nigella sativa* (black-cumin), *Hibiscus sabdariffa* (roselle), and *Origanum vulgare* (oregano) have received considerable interest based on scientific evidence. Beside these dietary plants, phytochemicals—such as catechin, epicatechin, epigallocatechin-3-gallate, diosmin, rutin, quercetin, hyperoside, and curcumin—as antioxidant dietary phyto-phenols were found to be effective for the prevention of urolithiasis (the process of stone formation in the urinary tract). The main underlying mechanisms of these dietary plants and their isolated phytonutrients in the management of urolithiasis include diuretic, antispasmodic, and antioxidant activity, as well as an inhibitory effect on crystallization, nucleation, and aggregation of crystals. The results as presented in this review demonstrate the promising role of dietary plants and phytophenols in the prevention and management of kidney stones. Further investigations are required to confirm the safety and efficacy of these compounds.

## 1. Introduction

Kidney stones, the formation of stones in the kidneys, is one of the oldest known and widespread diseases in the urinary tract system with a relapse rate of 50% in 5–10 years [1,2]. It is the third most common disorder among urinary diseases [3]. It has been reported that 10–12% of people in industrialized countries (10% of men and 3% of women) have a urinary stone during their lives. The etiology of this disorder is multifactorial and is related to genetics, diet, and low activity [4,5]. Calcium-containing stones are the most common kidney stones (75–90%), followed by magnesium ammonium phosphate (struvite) (10–15%), uric acid (3–10%), and cystine (0.5–1%) [6]. The mechanisms related to the development of kidney stones are not completely understood. Generally, it is believed that urolithiasis, the process of stone formation in the urinary tract, causes crystal aggregation, nucleation, and growth of insoluble particles [7]. The stones may cause various symptoms, including pain, obstruction, infection, and hemorrhage, through the passage of stones in the urinary tract system [8]. Treatment and management of renal stones relies on surgical techniques, such as extracorporeal shock wave lithotripsy, percutaneous lithotripsy, and transureteral lithotripsy [9,10]. These surgeries are complex and expensive and do not affect the recurrence of stones [9]. Various medicines, including thiazide as diuretic and alkali-citrate, are applied to prevent the frequency of hypercalciuria and hyperoxaluria—which cause calculi formation—but they are not promising enough due to their limited effectiveness and low tolerability [10,11,12,13]. Because of the disadvantages of surgical techniques and limited choice in pharmacotherapy, exploring new pharmacological therapies for the management of kidney stones is worthwhile. Various medicinal plants with diuretic, antispasmodic, and antioxidant activities exert inhibitory effects on crystallization, nucleation, and aggregation of crystals, making them useful for treatment of urolithiasis. The aim of the present article is to provide a critical review of the role of dietary plants as natural supplements in the prevention or management of kidney stones and elaborate underlying pharmacological mechanisms as well as their phytochemical constituents responsible for this activity.

## 2. Literature Search Methodology

Electronic databases, including PubMed, Science Direct, and Scopus, were searched for dietary plants and their bioactive compounds used for prevention and management of urolithiasis from 2005 to December 2017. The keywords were “kidney stone” or “urolithiasis”, or “nephrolithiasis”, or “renal calculi”, or “renal stone”, or “antilithiatic”; and “dietary plant”, or “dietary herb”, or “phytochemical”, or “fruits”, or “vegetables”. The retrieved articles were subclassified into in vitro, in vivo, and clinical studies. The studies included were evaluated with respect to the potential of the plant to be used as a dietary agent, the phytochemical composition of the plant, the kind of kidney stone that the dietary agent is effective on, as well as underlying mechanisms of action.

## 3. Role of Natural Diet in the Prevention of Kidney Stones

Emerging human studies have suggested that diets with a higher intake of vegetables and fruits play a role in the prevention of urolithiasis [14,15,16]. Epidemiological studies showed that diet could be one of the main risk factors for kidney diseases. Small-scale human investigations reported that diets with greater ingestion of plant-sourced protein, in comparison with animal-sourced protein, can cause amelioration of metabolic acidosis—attenuating further nephropathy progression in patients with chronic kidney disease—and reduction of glomerular filtration rate (GFR). Such dietary interventions, focusing on acid lessening with sodium-based alkalis, are among the main protective strategies in patients with reduced GFR [17]. It has been found that regular intake of natural diets rich in plants can increase urine pH and volume, as well as the amounts of stone inhibitors such as phytate, citrate, potassium, and magnesium, which are associated with supersaturation of calcium oxalate and uric acid [18]. Phytate is the main form of phosphate in natural sources, and its dietary intake is associated with the development of insoluble complexes with calcium within the gut, which can cause suppression of crystal formation in the urine and decrease the risk of urolithiasis [14,19]. Alkali load induced by a natural diet is able to raise urinary citrate which has a significant preventive effect on the development of kidney stones [20]. Additionally, dietary fiber which is abundant in fruits and vegetables can diminish the formation of stones due to its non-digestible ingredients which link to minerals and fat within the gastrointestinal tract, resulting in the suppression of urinary excretion of oxalate and calcium [14]. A human study evaluating the association between intake of dietary plants, fruits, and vegetables and the risk of incidence of urolithiasis in female subjects showed no history of kidney stones. These relationships were evaluated with stone recurrence in women with a history of kidney stones and it has been found that higher intake of fruits and vegetables was related to a decreased risk of development of urolithiasis [21].

## 4. Dietary Plants for the Prevention of Kidney Stones

Dietary interventions are taken into account as promising methods for kidney protection, either in concert with, or apart from, inherited or genetic factors. Nutritional plants are efficient remedies in the diet, which can influence risk of recurrence in calcium oxalate stones. In the following section, we discuss various dietary plants, food additives, fruits, and vegetables with well-established protective effects on urolithiasis. The details of the prophylactic roles of these plants on renal stones are also presented in Table 1.

### 4.1. Green Tea

Green tea (*Camellia sinensis*) has long been used as an herbal remedy with several polyphenols making them potent antioxidants. Although it is an oxalate-rich natural agent and could not be recommended for renal calculi formed by calcium oxalate [41], due to the anti-lithogenic, anti-atherosclerotic, and antioxidant effects of green tea, it has received considerable attention for use as a dietary supplement in patients suffering from nephrolithiasis and urinary stones [22,23,41,45]. The protective effect of green tea is most likely due to the presence of polyphenols and other phytochemicals. Green tea catechins, including epigallocatechin gallate (EGCG), epigallocatechin (GGC), epicatechin gallate (ECG), and epicatechin (EC), provide protective effects against oxalate-induced toxicity [23,41]. Green tea supplementation inhibited the growth of crystals in kidney of rats, diminished the excretion of oxalate [24,41], and exerted inhibitory effects on the activities of γ-glutamyltranspeptidase and *N*-acetyl-β-d-glucosaminidase [41,45]. It decreased the supersaturation of brushite [45], down-regulated the osteopontin (OPN) protein expression, increased superoxide dismutase (SOD), elevated Bcl-2 expression, and decreased the apoptotic index in the rat model of kidney stones [23]. These results, altogether, demonstrated that green tea rich in antioxidants possesses a protective effect against development of calcium stones in the kidneys.

### 4.2. Raspberry

Raspberry (Rubusidaeus, from Rosaceae family), is a commercial fruit crop grown in many European and Mediterranean countries and has been widely used for nutritional and medicinal purposes [46]. Raspberry has been found to be capable of expelling stones from the urinary tract even after acute administration. The prophylactic effect of raspberry on calcium oxalate renal stone formation has been reported [24]. Its aqueous extract exerted significant preventive effects on the deposition and precipitation of calcium oxalate in the kidney and eliminated the calcium oxalate matrix. The generation of malondialdehyde (MDA) and protein carbonyls was suppressed in raspberry-treated animals with decreased levels of urinary calcium and phosphorus. The presence of polyphenols and alteration in the level of stone formation inhibitors (such as citrate, magnesium, and glycosaminoglycans) may be involved in the mechanism by which raspberry inhibited the growth of calculus [24]. The methanolic extract of raspberry was found to be a potent diuretic via inhibiting the activity of aldosterone or epithelial sodium channels [25].

### 4.3. Rubia cordifolia

*Rubia cordifolia* (madder or Indian madder) belongs to the coffee family (*Rubiaceae*) and has been used as a natural food colorant. Phytochemical screening of *R. cordifolia* has revealed the presence of various bioactive phytochemicals, including glycosides, triterpenoids, anthraquinones, saponins, quinones, and tannins, which make it advantageous for the treatment of several disorders, such as jaundice, diabetic foot ulcer, and cardiovascular ailments [47,48]. It was found to be effective in the treatment of different kidney diseases and possessed preventive effects on urinary stones [26,49,50,51]. The hydro-alcoholic extract of *R. cordifolia* roots successfully inhibited the excretion of calcium in ethylene glycol-induced renal stone formation in rats, and, more importantly, prevented hyperoxaluria and hypocitraturia by decreasing the formation of urinary oxalate and regulating the re-adsorption of tubular citrate (increasing the level of urinary citrate), respectively. The preventive role of this extract against kidney stone formation was also due to its inhibitory effect on the deposition and growth of calcium oxalate crystals by restoring magnesium levels, its preventive effect on proteinuria, and its suppressive effect on acid uric excretion. The nephro-protective effect of this medicinal and nutritional plant could be largely attributed to its antioxidant properties [26].

### 4.4. Parsley

Parsley (*Petroselinum crispum*) belonging to the family *Umbelliferae* is commonly known as an herb, spice, and vegetable, and is widely distributed in Western Asia, the Mediterranean, and several European countries [52,53]. Various pharmacological activities, such as antioxidant, anti-inflammatory, diuretic, nephro-protective, enzyme-modulatory, and anti-hypertensive actions, have been reported for this plant [52,54]. These beneficial activities could be due to its bioactive constituents, including flavonoids, carotenoids, coumarins, tocopherol, and ascorbic acid [55]. Parsley and its extracts have been used potentially as a complementary/alternative treatment for various renal diseases [55,56,57]. *P.crispum* has been used as a promising anti-urolithiasis remedy. Its ethanolic extract prevented the nucleation and precipitation of calcium oxalate, urine supersaturation, and urinary protein excretion in a rat model of calcium stone formation [27]. The high content of chlorophyll and magnesium in parsley is a reason for its inhibitory effect toward the dehydration of calcium oxalate and hyperoxaluria, respectively [58]. Parsley was found to be effective in regulating urinary pH at a value at which calcium oxalate crystals could be maintained as dispersed particles, and the elimination of these crystals could be facilitated [27,28].

### 4.5. Pomegranate (Punica granatum)

Pomegranate has long been used in traditional medicine. Pomegranate fruit, known as “a pharmacy unto itself” [59], is a rich source of polyphenols, alkaloids, and anthocyanins (flavonoid antioxidants), which are highly capable of scavenging free radicals [60,61]. All parts of this plant can be used in traditional remedies for preventive and therapeutic purposes. Pomegranate seeds were used for regulating urine discharge and the burning sensation of urine; its seed oil, juice, flowers, and peel are used for protection against nephrotoxicity [62,63,64,65], and the extracts for renal failure [66] and renal arteries [67]. The anti-hypercalciuria and anti-urolithiasis effects of this plant attracted considerable attention toward pomegranate for use in the prevention of renal calculus formation. Its therapeutically beneficial phytochemicals are responsible for muscle relaxation in the urinary and biliary tract; consequently, stones can be easily removed from the kidney [12]. Administration of the methanolic extract of pomegranate to the rat model of urolithiasis (induced by 28 days of treatment with ethylene glycol) dose-dependently inhibited the inflammation mediated by ethylene glycol, and consequently regulated the levels of oxalates, calcium, and phosphates. The methanolic extract was also found to be more protective in comparison with the chloroform extract, which might be due to the lipophilic nature of pomegranate constituents [12]. The extracts and juice of pomegranate significantly inhibited the hyperoxaluria-induced oxidative renal tubular damages (due to its antioxidants and anti-lipid-peroxidation [68]) by reducing the levels of reactive oxygen species (ROS), inducible nitric oxide synthase (iNOS), and nuclear factor-κB (NF-κB) [61,69] and p38-mitogen-activated protein kinase (p38-MAPK) [69], and regulating urea, creatinine, and ureic acid [12]. Beside the animal studies, the nephro-protective roles of pomegranate extract on the calcium-containing lithiasis formation in humans (18–70 years old) with recurrent stone formation have been clinically studied. The daily supplementation of patients with pomegranate extract caused significant down-regulation of serum paraoxonase1 (PON1) arylesterase activity together with decreasing supersaturation of calcium oxalate, indicating that this intervention could successfully control the risk of renal stone formation [42].

### 4.6. Pistacia lentiscus

*Pistacia lentiscus* (Anacardiacceae) is a common evergreen dioecious shrub, distributed in a wide range of habitats, specifically in the eastern Mediterranean region [70]. In folk medicine, it is known as a medicinal and nutritional plant with various therapeutic potentials, such as antioxidant, anti-microbial, diuretic, anti-lipid peroxidation, and anti-urolithiasis activities [70,71]. The fruit extract has demonstrated in vitro potential in protecting human kidney (HK)-2 cells against proximal tubular injury mediated by calcium oxalate monohydrate (COM). It significantly inhibited the cell death induced by COM and suppressed the level of E-cadherin, as well as H_2_O_2_. It attenuated the attachment and internalization of calcium oxalate monohydrate crystals to epithelial tubular cells by the mechanism in which the interaction of active phytochemicals of the extract (mainly polyphenols) with cells inhibited its binding to the surface of the cells [38]. Therefore, *P. lentiscus* could be considered as a promising natural remedy for antilithiatic purposes.

### 4.7. Solanum xanthocarpum

*Solanum xanthocarpum*, also known as “yellow-fruit nightshade” and “Thai green eggplant”, is a famous and widely used edible traditional medicinal plant in India. The seeds and fruits are consumed as foods and vegetables [72]. This plant is used as a common remedy for the treatment of various renal diseases, including difficulty in urination, urinary infections, nephrotoxicity, and urolithiasis [30,73,74,75]. The fruit of *S. xanthocarpum* is a rich source of steroidal glycol-alkaloids, coumarins, triterpenes, and saponins [30]. The petroleum-ether extract of the fruits exhibited nephro-protective activity, possibly due to the anti-lipid peroxidation and antioxidant effects of the plant constituents [75]. The methanolic extract was found to be successful in preventing and inhibiting nephrolithiasis, renal hyperoxaluria, crystalluria, and supersaturation of calcium oxalate. It exerted antioxidant (by increasing SOD and glutathione (GSH) levels) and diuretic activities and also diminished the excretion of phosphorous in the calculi-induced rats [30]. The fruits of this plant contain saponins with high antilithiatic activity. The saponin-rich fraction prepared from fruits of *S. xanthocarpum* showed prevention of in vitro calcium oxalate crystal nucleation and aggregation in artificial urine solution, and inhibition of pathological changes due to lithogenic treatment, including polyuria, damage of renal function, oxidative stress, and crystalluria in ethylene glycol-induced urolithiasis in rats. The aforementioned fraction also increased levels of glycosaminoglycan, a stone inhibitor macromolecule found in urine, and accelerated the glomerular filtration [30].

### 4.8. Urtica dioica

*Urtica dioica* or “Stinging Nettle”, which belongs to the nettle genus of Urticaceae family, is used as tea in Austrian medicine [31,76]. It has shown a long history of beneficial therapeutic effects toward urinary ailments, specifically with the urinary tract and kidney stones. Its major bioactive phytochemicals include flavonoids, anthocyanins, and saponins [31]. These phytoconstituents provide the possibility of inhibition of calcium and oxalate deposition and crystals growth. Supplementation of the methanolic extract *U. dioica* to rats with kidney stones (induced by ethylene glycol and ammonium chloride) was found to be associated with decreased urinary creatinine level and reduction of supersaturation of lithogenic enhancing agents. This extract potentially dissolved the lithiasis and overcame the hyperoxaluria and crystalluria induced by ethylene glycol [31].

### 4.9. Dolichos biflorus

*Dolichos biflorus* (*horse gram*) is a nutritional and medicinal plant native to India, where its seeds are used to prepare soup [77]. The seeds are acclaimed in ayurvedic literature to have litholytic, free radical-scavenging, and anti-nephrotoxic effects [40,43,77]. The beneficial effect of this plant can be attributed to the existence of various phytoconstituents in the seeds, including phenolic compounds (such as quercetin), alkaloids, phytosterols (such as β-sitosterol), saponins, glucosides (such as β-galactosidases and α-mannosides) [39,40]. Various extracts from seeds, including aqueous, chloroform, and benzene, dissolved calcium oxalate stones in experimental models of kidney stones. Aqueous extract showed the highest dissolution of stones compared to other extracts [40]. In a synthetic urine system for calcium oxalate crystallization, the hydro-alcoholic extract of seeds showed inhibitory activity on nucleation and aggregation of calcium oxalate monohydrate crystals [39]. Administration of *D. biflorus* to patients with calcium oxalate renal calculi decreased the recurrence of calcium oxalate stones and had a better result than the use of potassium citrate in these patients [43].

### 4.10. Ammi visnaga

Teas prepared from the fruits of *Ammi visnaga* have been traditionally used by patients with renal stones in Egypt [32]. The aqueous extract of this fruit accelerated the dissolution of cystine stones [37]. The fruit and its two major constituents, namely khellin and visnagin, showed beneficial effects in the management of kidney stone disease caused by hyperoxaluria in male rats through reduction of the incidence of calcium oxalate crystal deposition, increasing urinary excretion of citrate along with a decrease of oxalate excretion [32].

### 4.11. Nigella sativa

*Nigella sativa* has been used in Iranian traditional medicine for treatment of urinary stones [33,34,78,79]. Ethanolic extract of seeds reduced the number of calcium oxalate deposits in ethylene glycol-induced lithiatic rats and decreased the urine concentration of calcium oxalate [33]. Thymoquinone, the major component of the seeds, showed preventive and therapeutic effects on ethylene glycol-induced kidney calculi in rats. This phytochemical compound decreased the size and number of calcium oxalate deposits in the renal tubules in rats [34].

### 4.12. Hibiscus sabdariffa

Based on Thai traditional medicine, *Hibiscus sabdariffa* is used for the prophylaxis and treatment of urinary stones [44]. It has been found that the main active constituents of this plant include polyphenols, hibiscus anthocyanins, as well as L-ascorbic acid, quercetin, and protocatechuic acid. The aqueous plant extract had demonstrated antiurolithiatic activity due to the decreased deposition of stone-forming constituents in the kidneys and serum of ethylene glycol-induced urolithiatic rats [36]. Moreover, the plant extract had an antilithic effect on rats on a glycolate diet through the decrease in oxalate retention time in the kidneys and more excretion into urine [80]. A clinical trial—which had tested a cup of tea made from 1.5 g of dry *H. sabdariffa* two times daily on 18 patients for 15 days—revealed uricosuric effect and significant increase in uric acid excretion and clearance [44].

### 4.13. Origanum vulgare

This plant has been widely used as spice and in traditional medicine as a lithotriptic, diuretic, and antispasmodic [11]. The crude aqueous-methanolic extract of the aerial part of *O. vulgare* exhibited in vitro inhibitory activity in the nucleation and aggregation of calcium oxalate crystals, and also decreased the number of crystals produced in calcium oxalate metastable solutions. Evaluation of rats with ethylene glycol and ammonium chloride-induced urolithiasis showed that the extract of the aerial part of *O. vulgare* had antiurolithic activity, possibly through prevention of calcium oxalate crystallization, renal epithelial cell protection, antioxidant, and antispasmodic properties. The preventive effect could be attributed to its active phytochemicals including flavonoids, terpenes, coumains, saponins, alkaloids, sterol, and tannins [11].

## 5. Medicinal Plants and Phytoconstituents as Dietary Supplements for the Prevention of Kidney Stones

In current years, there is great interest in herbal and traditional medicine for prevention and management of variety of diseases [81]. Medicinal plants have been used for thousands of years for the prevention of the development and recurrence of kidney stones in different countries [82,83]. Various medicinal plants and phytochemical constituents have been evaluated for their preventive and therapeutic potential in kidney stones [84]. Medicinal plants with well-established preclinical and/or clinical evidence of their protective or therapeutic effect in urolithiasis include *Bergenia ciliata* (Haw.) Sternb [85], *Bergenia ligulata* Engl. [86,87], *Commiphora wightii* (Arn.) Bhandari [88], *Costus arabicus* L. [89], *Herniaria hirsuta* L. [90], *Terminalia chebula* Retz. [91], *Tribulus terrestris* L. [92], *Acalypha indica* L. [93], *Aerva lanata* (L.) Juss. [94], *Ageratum conyzoides* (L.) L. [11], *Alcea rosea* L. [95], *Asparagus racemosus* Willd. [96], *Bombax ceiba* L. [97], *Carthamus tinctorius* L. [98], *Cynodon dactylon* (L.) Pers. [99], *Helichrysum graveolens* (M. Bieb.) Sweet and *Helichrysum stoechas* ssp. barellieri (Ten.) Nyman [100], *Hordeum vulgare* L. [101], *Hygrophila spinosa* T.Anderson [102], *Hypericum perforatum* L. [103], *Launaea procumbens* L. [104], *Lygodium japonicum* (Thunb.) Sw. [105], *Orthosiphon grandiflorus* Bold. [106], *Paronychia argentea* Lam. [107], *Pedalium murex* L. [9], *Pergularia daemia* (Forssk.) Chiov. [108], *Quercus salicina* Blume [109], *Salvadora persica* L. [110], *Selaginella lepidophylla* (Hook. et Grev) Spring [111], *Agropyron repens* (L.) P.Beauv. [112], and *Phyllanthus niruri* L. [113].

These nephro-protective herbs, were found to be effective inhibitors of the formation and growth of calcium hydrogen phosphate dihydrate (Brushite) crystals, calcium hydrogen phosphate dehydrate (CHPD) crystals, calcium oxalate monohydrate crystals, and cysteine and uric acid stones [85,86,87,101,104,114,115].

The details on the study design and pharmacological evidences of these medicinal plants are presented in Table 2 and Table 3.

## 6. Effect of Pharmacologically Active Phytochemicals on the Inhibition of Urolithiasis

Several recent studies have highlighted the effectiveness of dietary interventions as a promising method for kidney protection, either in concert with, or apart from, inherited or genetic factors. Nutritional plants and their phytochemicals could be included either in the main diet or as dietary supplements to treat urolithiasis, reduce the risk of recurrence of kidney stones, and affect the formation and growth of crystals. Several medicinal herbs and natural compounds have been successfully applied for these purposes, while the molecular basis underlying the prophylactic effect of these phyto-therapeutics is poorly understood. The anti-urolithiasis effects of nutraceuticals have been thought to be most likely due to their antioxidative effects. Calcium oxalate is the major constituent of urolithiasis, and antioxidant therapy could be one of the effective methods for preventing the nucleation as well as binding and growth of calcium oxalate crystals. Dietary phyto-phenols, with more than 8,000 structural variants, are the most abundant antioxidants in human diets which can occur in a variety of vegetables and fruits [116]. These nutraceuticals exhibited high inhibitory effects against the oxidative stress-associated dysfunctions in kidney (Table 4). Catechin and epicatechin are two of these antioxidants mostly included in plant sources, such as tea (green and black) and grape seeds [117]. The antioxidant activity of catechin could be attributed to either its radical scavenging and metal-chelating properties, or its modulatory effect on transcription factors and enzymes [118]. This provides the reno-protective capability toward different renal injuries, oxidative stress associated with renal failure, and renal calcium crystallization [119,120]. Catechin increased the SOD activity in COM-treated NRK-52E renal proximal tubular cell line and restored the mitochondrial membrane potential and cleavage of caspase-3 [121]. The renal papillary calcification and enhancement of COM papillary calculi have been successfully inhibited by catechin [122], which could be due to its promoting effect on SOD activity [119]. The OPN, MDA, and 8-hydroxy-2′-deoxyguanosine (8-OHdG) were also well-regulated by catechin in the ethylene glycol-induced rat model of nephrolithiasis [121]. EGCG, as an important catechin, also exerted the protective effect by attenuating the binding capability of Madin–Darby canine kidney (MDCK) cells to COM crystals. This was found to be attributed to the reduced α-enolase protein expression (responsible for binding) on the renal tubular cell surface following EGCG administration [123].

Diosmin, as a flavonoid glycoside, is another polyphenol with inherent antiurolithiatic activity which can be found in vegetables and citrus fruits [124]. It has been commonly used as a natural medicament for various renal diseases and protects kidneys from diabetic nephropathy, nephro-toxicity, and oxidative stress [125,126,127,128]. The renoprotective effects of diosmin were found attributable to its suppressing activity on lipid peroxidation, potentiating activity on antioxidant enzymes, and its modulatory effect on the expressions of Bax and p53 proteins [127]. Beside these properties, it exhibited the potential to block the process of calcium oxalate stone formation in the rat model of nephrolithiasis. It exerted its antiurolithiatic effect by diminishing the capillary hyper-permeability, attenuating the degeneration of glomeruli and tubules, and restoring the diameter of the capillaries and vessels in the cortex [129]. Diosmin could also prevent the crystallization of stone-forming promoters by keeping the urinary pH at acidic values. This increased urine volume, elevated urinary level of potassium and magnesium, and suppressed level of urinary protein [130]. Rutin, quercetin, and hyperoside, like diosmin, are known as flavone glycosides with high antioxidant and anti-lithiatic activities. Rutin can be found in variety of plants, and black tea and apple peels are common dietary sources of this nutraceutical [131]. Rutin therapy, alone or in combination with curcumin, was found to be a successful remedy for prevention of stone formation [132,133]. Co-administration of these two phyto-phenolic compounds in calculi-induced rats caused restoration of the urinary calcium and oxalate levels and attenuated lipid-peroxidation. The aggregation and growth of COM crystals and glomerular filtration rate were found to be affected by these phyto-phenols [133]. Besides the antioxidative potential, the anti-inflammatory effect of these phytochemicals could be, in part, included in their prophylactic mechanism. Quercetin and hyperoside (mostly found in vegetables and fruits) have also exhibited promising antioxidant, diuretic, hypo-uricemic, and anti-inflammatory activities [134]. These two bioflavonoids are taken into account as efficient phyo-therapeutics for management of renal lithiasis based on their inhibitory effect on the deposition of calcium oxalate crystals, antioxidant activity against renal tubular cell injury (via increasing SOD and catalase activities), and anti-apoptotic effects [134,135]. The ability of quercetin to promote serum PON1 also provided an efficient antioxidant effect on hyperoxaluria-induced rats [119].

## 7. Major Pharmacological Mechanisms of Plants and Natural Products in the Prevention of Kidney Stones

Medicinal plants could affect different aspects of urolithiasis pathophysiology [136]. The medicinal plants can be effective in prophylaxis, treatment, and prevention of kidney stone recurrence. Their mechanisms of actions include increasing excretion of urinary citrate, decreasing excretion of urinary calcium and oxalate, inhibition of the nucleation and growth of the calcium oxalate crystals, dissolving stones, raising the level of glycosaminoglycan, and being diuretic. In addition to the hypermagneseuric inhibitory effect on crystallization and aggregation of crystals, cytoprotective, nephroprotection, antioxidant, and antispasmodic pharmacological effects of dietary natural components are among other mechanisms involved in protection against urolithiasis.

**Figure 1:**
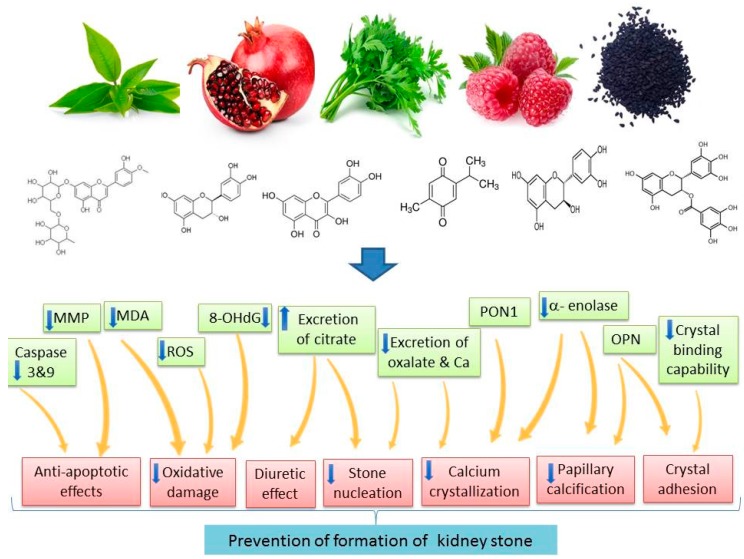
Main mechanisms of action of dietary polyphenols in the prevention of kidney stones (Up arrow demonstrates increasing trend; down arrow demonstrates decreasing trend).

Recurrence of kidney stones is an important problem. Studies have revealed that treatments like phyto-medication or dietary modification can reduce the recurrence rate. Therefore, recurrence of renal stones is partially preventable [9]. Drugs, including thiazide as diuretic and alkali-citrate, are being used to prevent the recurrence of hypercalciuria and hyperoxaluria, but evidence for their efficacy is low [12]. Medicinal plants can prevent recurrence of renal calculi by lithotriptic activity, regulation of oxalate metabolism, modulating the crystalloid colloid imbalance, and decreasing supersaturation which inhibits crystallization. For example, *B.ligulata* rhizome extract has been reported to suppress calcium oxalate crystal precipitation through interference with crystal growth and aggregation [86]. The leaf extract of *Launaea procumbens* is effective in preventing the recurrence of renal calculi by its activity on early stages of stone development [104]. Moriyama et al. [43] demonstrated that *Quercus salicina* extract inhibited renal calcium accumulation and urinary MDA excretion in a rat model of calcium oxalate urolithiasis, possibly by reducing oxidative stress. Dietary plants and their isolated natural polyphenols were also effective natural remedies based on their prophylactic effects on urolithiasis. The preventive role of these nutritional plants and their polyphenols is attributed to their well-established pharmacological mechanisms in the kidney, including attenuation of hyperoxaluria, proteinuria, and hypocitraturia; their inhibitory effect on the deposition and the growth of calcium oxalate; down-regulation of serum PON1; up-regulation of antioxidant enzymes; and suppression of the attachment and internalization of calcium oxalate monohydrate crystals to epithelial tubular cells (Figure 1).

## 8. Concluding Remarks

Dietary plants, including food additives, fruits and vegetables, have a pivotal role in human health and the prevention of diseases, including kidney stones. However, the pharmacological evidence of dietary plants and their phytonutrients in the prevention of kidney stones have not been well-established yet. In this review, we have presented evidence on the efficacy and pharmacological mechanisms of various dietary plants and their phytochemicals in the prevention or management of kidney stones. Current literature showed that a large number of in vitro and animal studies were conducted to evaluate the preventive effects of dietary plants and their phytochemicals as dietary supplements in the development of urolithiasis. However, limited human studies on the efficacy of medicinal as well as dietary plants in the management of kidney stone were performed. *A. repens* (L.), *D. biflorus* L., *H. sabdariffa* L., *P.granatum* L., and *Phyllanthus niruri* L. are among the plants whose efficacy have been confirmed by clinical trials. Nutraceuticals (mainly dietary polyphenols), including catechin, epicatechin, EGCG, diosmin, rutin, quercetin, hyperoside, and curcumin, could be proposed as promising dietary supplements for prevention of urolithiasis.

To conclude, results obtained from the available literature revealed that dietary plants and their phytonutrients could be useful in the prevention and intervention of urolithiasis. Since natural dietary recommendations for patients with the risk of kidney stones are poorly provided, and patients often request instructions for a beneficial dietary regimen, it is essential for physicians to have evidence-based knowledge regarding the efficacy, pharmacological mechanisms, and side effects of the administration of a protective dietary regimen. More investigations using clinical trials are needed to confirm the efficacy and safety of these dietary agents in patients with kidney stones. Moreover, to achieve more conclusive results, further preclinical and human studies are compulsory to reveal molecular and cellular mechanisms, as well as the bioactive phytonutrients of these dietary plants in urolithiasis.

## Figures and Tables

**Table 1:** Experimental and clinical evidence on nutritional plants used for prevention and treatment of kidney stones.

Plant	Part of Plant	Study Type	Study Design	Results	Reference
Green tea (*Camellia sinensis*)	Leaves of kidney stones	In vivo	Ethylene glycol (EG)-induced nephrolithiasis in rat	↓ Calcium crystal depositions in the kidneys ↓ The osteopontin mRNA level	[22]
	Leaves	In vivo	EG-induced nephrolithiasis in rat	↓ Urinary oxalate excretion, calcium oxalate deposit formation ↑ Sodium Oxide Dismutase (SOD) activity	[23]
Rasberry (*Rubus idaeus*)	Aqueous extract	In vivo	Glyoxylate-induced calcium oxalate (CaOx) nephrolithiasis in mice	↓ Generation of malondialdehyde (MDA) and protein carbonyls ↓ Urinary calcium and phosphorus levels ↓ The growth rate of calculus	[24]
	Methanolic extract	In vivo	Bicarbonate saline solution (containing 110 mM NaCl and 30 mM NaHCO3) induced nephrolithiasis in rats	↓ Activity of aldosterone or epithelial sodium channels ↑ Urine volume	[25]
Common madder (*Rubia cordifolia*)	Hydro-alcoholic extract	In vivo	EG-induced urolithiasis	↓ The growth of calcium oxalate crystals ↓ The formation of urinary oxalate ↑ Tubular citrate	[26]
Parsley (*Petroselinum sativum* Hoffm.)	Ethanolic extract	In vivo	EG+ ammonium chloride (AlCl3)-induced urolithiasis in rat	↓ Urinary calcium and protein excretion ↑ Urinary pH	[27]
	Aqueous Extract	In vivo	EG-induced urolithiasis in rats	↓ Serum urea and uric acid concentrations ↑ Serum magnesium concentration	[28]
Parsley (*Petroselinum sativum* Hoffm.)	Aerial parts and roots aqueous extract	In vivo	EG-feeding rats	↓ The number of calcium oxalate deposits	[29]
Pomegranate (*Punica granatum*)	Fruits chloroform and methanol extract	In vivo	EG-induced urolithiasis	↓ Urine oxalate, calcium and phosphate, renal tissue oxalates ↓ Serum creatinine, urea and uric acid	[24]
Yellow-fruit nightshade (*Solanum xanthocarpum*)	The methanolic extract	In vivo	EG-induced urolithiasis in rats	↓ Renal hyperoxaluria and crystalluria, ↓ Supersaturation of calcium oxalate	[30]
Stinging nettle (*Urtica dioica*)	Methanolic extract	In vivo	EG-induced urolithiasis in rats	↓ Urinary creatinine level and the supersaturation of lithogenic enhancing agents	[31]
*Khella* (*Ammi visnaga* L.)	Aqueous extract of fruits	In vivo	EG+ aluminum chloride-induced urolithiasis in rats	↓ Calcium oxalate crystal deposition ↑ Urinary excretion of citrate ↓ Oxalate excretion	[32]
Black-cumin (*Nigella Sativa* L.)	Ethanolicextract of seeds	In vivo	Ethylene glycol for induction of calcium oxalate calculus formation in rats	↓ Number of calcium oxalate deposits ↓ Urine concentration of calcium oxalate	[33]
	Thymoquinone (major component of seeds)	In vivo	Ethylene glycol-induced kidney calculi in rats	↓ Number and size of calcium oxalate deposits in the renal tubules	[34]
*Citrus aurantium* L.	Aqueous extract of unripe fruit	In vivo	EG -induced calcium oxalate crystallization	Preventing the formation of calcium oxalate nephrolithiasis and pathological alterations in rats	[35]
Oregano (*Origanum vulgare* L.)	Aerial part aqueous-methanolic extract	In vivo	EG-induced urolithiasis in rats	Preventing loss of body weight, polyurea, crystalluria, oxaluria ↑ Serum urea and creatinine levels	[34]
*Roselle* (*Hibiscus sabdariffa* L.)	Plant aqueous extracts	In vivo	EG-induced hyperoxaluria	↓ Deposition of stone-forming constituents in the kidneys and serum	[36]
*Khella* (*Ammi visnaga* L.)	aqueous extract	In vitro	A flask containing a cystine stone	↑ Dissolution rate of cystine stones	[37]
*Mastic* (*Pistacia lentiscus*)	ethanolic fruit extract	In vitro	Calcium oxalate monohydrate-induced in Human Kidney (HK)-2 cells	↓ Cell death induced by COM, ↓ The level of E-cadherin and H_2_O_2_	[38]
Roselle (*Dolichos biflorus* L.)	Hydro-alcoholic extract of seeds	In vitro	Calcium oxalate crystallization using a synthetic urine system	↓ Nucleation and aggregation of calcium oxalate monohydrate crystals	[39]
	Aqueous, chloroform, and benzene extracts of seed	In vitro	Experimental preparation of kidney stones; calcium oxalate and calcium phosphate	Dissolving calcium oxalate stones	[40]
Oregano (*Origanum vulgare* L.)	Crude aqueous-methanolic extract	In vitro	Supersaturated solution of calcium oxalate, kidney epithelial cell lines (MDCK) and urinary bladder of rabbits	↓ Calcium oxalate crystallization Exerting antioxidant, renal epithelial cell protective and antispasmodic activities	[41]
*Solanum xanthocarpum*	Saponin rich fraction prepared from fruits	In vitro	calcium oxalate crystal nucleation. artificial urine solution	↓ Calcium oxalate crystal formation ↑ Glycosaminoglycan level	[42]
Pomegranate (*Punica granatum*)	Extract capsule	Clinical	23 recurrent stone formers (RSFs) and 7 non-stone formers (NSFs) (1000 mg daily) for 90 days	↓ Serum paraoxonasearylesterase activity ↓ Supersaturation of calcium oxalate	[43]
*Horse gram* (*Dolichos biflorus* L.)	Seed	Clinical	24 patients received *Dolichosbiflorus* and 23 patients were given potassium citrate	↓ Recurrence of calcium oxalate stone	[44]
*Roselle* (*Hibiscus sabdariffa* L.)	A tea bag of dried plant	Clinical	9 patients with renal stones and 9 with non-renal stone received tea (A cup of tea made from 1.5 g of dry herb two times daily	↑ Uric acid excretion and clearance	[45]

↑ demonstrates increasing trend; ↓ demonstrates deccreasing trend.

**Table 2:** Cellular studies on medicinal plants used for the prevention and treatment of kidney stones.

Plant	Part or Chemical Constituents	Study Type	Study Design	Results	Reference
*Bergenia ciliata* (Haw.) Sternb	Hydro-alcoholic extract of rhizomes	In vitro	Calcium oxalate induced in a synthetic urine system	↑ Nucleation and aggregation of COM crystals ↓ The number and size of COM crystals	[85]
*Bergenia ligulata* Engl.	Aqueous-methanolic extract of rhizome	In vitro	Calcium oxalate induced crystal in a synthetic urine system	Inhibition of crystal aggregation and formation ↑ Radical scavenging ability and lipid peroxidation	[86]
*Commiphora wightii* (Arn.) Bhandari	Extract	In vitro	Struvite crystals induced using gel growth technique	↓ Growth and the size of the struvite crystals	[88]
*Costus arabicus* L.	Aqueous dried plant extract	In vitro	Calcium oxalate monohydrate (COM) crystals induced in MDCK cells	↓ Crystal growth and calculogenesis	[89]
*Herniaria hirsuta* L.	Ether and methanol extracts of aerial parts	In vitro	Calcium oxalate-induced stone in urine	↓ The size and supersaturation rate of crystals	[90]
*Terminalia chebula* Retz.	Aqueous fruits extract	In vitro	Calcium oxalate induced cell injury in NRK-52E and MDCK renal epithelial cells	↓ Lactate dehydrogenase release ↑ Cell viability	[91]
*Tribulus terrestris* L.	Protein biomolecules	In vitro	Oxalate induced injury on NRK-52E cells	↓ Lactate dehydrogenase release ↑ Cell viability	[92]

↑ demonstrates increasing trend; ↓ demonstrates deccreasing trend.

**Table 3:** Pre-clinical and clinical evidence on medicinal plants used for prevention and treatment of kidney stones.

Plant	Part or Chemical Constituents	Study Type	Study Design	Results	Reference
*Acalypha indica* L.	Ethanolic extract	In vivo	Ethylene glycol (EG)-induced urolithiasis in Wistar albino rats	↑ Ca^2+^ ATPase, Mg^2+^ ATPase, Na^+^K^+^ ATPase ↑ Aspartate Transaminase (AST), Alanine Transaminase (ALT), Acid phosphatase (ACP) and Alkaline Phosphatase (ALP)	[93]
*Aerva lanata* (L.) Juss.	Aqueous suspension of aerial parts	In vivo	EG-induced urolithiasis in rats	↓ Glycolic acid oxidase (GAO), and lactate dehydrogenase (LDH)	[94]
*Ageratum conyzoides* (L.) L.	Hydroalcolohlic extract of whole plant	In vivo	EG-induced urolithiasis in rats	↓ Stone forming constituents, Blood urea nitrogen (BUN), uric acid and creatinine	[11]
*Alcea rosea* L.	Hydroalcoholic extract of roots	In vivo	EG-induced lithiasis in rats	↓ The number of calcium oxalate deposits ↓ Urinary oxalate level	[95]
*Asparagus racemosus* Willd.	Ethanolic extract of tuberous roots	In vivo	EG-induced urolithiasis in rats	↓ The level of calcium, oxalate, phosphate, and serum creatinine; ↑ Urinary concentration of magnesium	[96]
*Bergenia ciliata* (Haw.) Sternb.	The hydro-methanolic extract of rhizomes	In vivo	EG-induced urolithiasis in rats	↓ Nucleation and aggregation of crystals ↓ The number and size of COM crystals	[85]
*Bergenia ligulata* Engl.	Aqueous-methanolic extract of rhizome	In vivo	EG-induced urolithiasis in rats	↓ Calcium oxalate crystal deposition, and lithogenic signs ↑ Urinary magnesium	[96]
	Ethanolic extract of rhizome; bergenin	In vivo	EG+ aluminium chloride-induced urolithiasis in rats	↓ MDA level, ↑ H_2_O_2_ scavenging ability ↑ SOD, Catalase (CAT) and GP levels	[87]
*Bombax ceiba* L.	Fruit aqueous and ethanol extract	In vivo	EG-induced urolithiasis in rats	↓ Urinary oxalate ↓ Stone forming constituents	[97]
*Carthamus tinctorius* L.	Commercial herbal powder- gastric gavage	In vivo	EG-induced stones in rats	↓ Deposition of calcium oxalate crystal	[98]
*Cynodon dactylon* (L.) Pers.	N-butanol and ethyl acetate extract of root	In vivo	EG-induced calculus in rats	Preventing calcium oxalate deposition ↓ The size of crystals	[99]
*Helichrysum graveolens* (M.Bieb.) Sweet and *Helichrysum stoechas* ssp. barellieri (Ten.) Nyman	Capitulum aqueous extract	In vivo	Sodium oxalate- induced urolithiasis in rats	↓ Formation and growth of crystals ↓ Urine oxalate and uric acid levels, ↑ Citrate level	[100]
*Hordeum vulgare* L.	Seeds ethanolic extract	In vivo	EG-induced urolithiasis in rats	↓ Stone forming constituents ↓ Lipid peroxidation ↑ SOD and CAT	[101]
*Hygrophila spinosa* T.Anderson	Methanolic extract of aerial parts	In vivo	EG-induced nephrolithiasis in rats	↓ Urinary oxalate ↓ Calcium and oxalate in kidney; ↑ Urinary magnesium	[102]
*Hypericum perforatum* L.	Hydroalcoholic extract of leaves	In vivo	EG+ ammonium chloride- induced stone in rats	↓ The size and number of calcium oxalate deposits	[103]
*Launaea procumbens* L.	Methanolic extract of leaves	In vivo	EG-induced urolithiasis in rats	↓ Urinary calcium, oxalate and phosphate excretion ↓ Creatinine and uric acid	[104]
*Lygodium japonicum* (Thunb.) Sw.	Ethanolic extract of spore	In vivo	EG-induced kidney calculi in rats	↓ Urinary calcium, oxalate and uric acid ↓ Kidney peroxides, and the number of oxalate deposits ↑ Urinary citrate levels	[105]
*Orthosiphon grandiflorus* Bold.	Aqueous extract of leaves	In vivo	EG-induced stones in rats	↓ Crystal deposits ↑ SOD and CAT	[106]
*Paronychia argentea* Lam.	Butanolic extract of aerial parts	In vivo	Sodium oxalate-induced lithiasis in rats	↓ Renal necrosis ↓ Serum creatinine and blood urea levels	[107]
*Pergularia daemia* (Forssk.) Chiov.	Whole-plant hydroalcoholic extract	In vivo	EG- induced kidney stone in rats	↓ Serum urea nitrogen, creatinine and uric acid levels	[108]
*Quercus salicina* Blume	Leaves aqueous extract	In vivo	EG and the vitamin D3 analog(α-calcidol)-induced urolithiasis in rats	↓ MDA and serum creatinine level ↓ Oxidative stress ↓ Calcium level in kidney	[109]
*Salvadora persica* L.	Aqueous and alcoholic extract of the leaves	In vivo	EG- induced urolithiasis in rats	↓ Urinary oxalate levels and deposition	[110]
*Selaginella lepidophylla* (Hook. et Grev) Spring	Chloroform extract of the plant	In vivo	EG and ammonium chloride- induced urolithiasis in rats	↑ Urinary flow rate, glomerular filtration rate (GFR) ↓ ROS and lipid-peroxidation ↓ Renal cortical organic anion transporter (OAT3) expression	[111]
*Agropyron repens* (L.) P.Beauv.	Extract	Clinical	Unblinded treatment to the patients (treatment group received potassium citrate + *Agropyrum repens* and control group recieved potassium citrate alone (100 mg/day for 5 month))	↓ Number and size of urinary stones ↓ Uric acid urinary secretion	[112]
*Phyllanthus niruri* L.	Extract	Clinical	150 patients received 1 to 3 extracorporeal shock wave lithotripsy sessions. After treatment 78 patients received extract and 72 were served as a control group (2 g/day for 3 month)	↑ Stone-free rate (stone-free defined as the absence of any stone or residual fragments less than 3 mm)	[113]

↑ demonstrates increasing trend; ↓ demonstrates deccreasing trend.

**Table 4:** Cellular and animal evidence on phytochemicals used for prevention and treatment of kidney stones.

Photochemical	In Vitro/In Vivo	Model	Result	Reference
Catechin	In vitro	Calcium oxalate monohydrate(COM)-induced NRK-52E cells	↑ SOD activity ↓ Mitochondrial membrane potential (MMP), Caspase-3 activity, and renal calcium crystallization	[121]
	In vivo	Ethylene glycol (EG) induced nephrolithiasis in rat	↑ OPN, ↓ MDA, 8-OHdG ↓ Renal calcium crystallization	[121]
	In vivo	EG-induced nephrolithiasis in rat	↓ Calcium oxalate monohydrate and Papillary calculus formation ↓ Renal papillary calcification	[122]
Epigallocatechin-3-gallate	In vitro	COM-induced Madin–Darby canine kidney (MDCK) cells	↓ α-enolase protein expression ↓ crystal-binding capability	[123]
	In vitro	Oxalate-induced NRK-52E cells	↓ Free-radical production	[41]
	In vivo	Oxalate-induced renal stone in rats	↓ Excretion of urinary oxalate ↓ Activities of urinary gammaglutamyl transpeptidase and N-acetylglucosaminidase	[41]
Diosmin	In vivo	EG-induced nephrolithiasis in rat	↓ Capillary hyper-permeability ↓ Degeneration of glomeruli and tubules, Restoring the diameter of the capillaries and vessels in the cortex	[129]
Rutin	In vivo	EG-induced nephrolithiasis in rat	Prevention of stone formation Inhibition of calcium oxalate urolithiasis	[133]
Quercetin	In vivo	EG induced calcium oxalate (CaOx) formation	Hypo-Uricemic, and anti-inflammatory activities Inhibitory effect on the deposition of CaOx crystal	[134]
	In vitro	Sodium oxalate	↓ Cell viability ↓ Lipid peroxidation	[135]
	In vivo	Hyperoxaluria-induced rats	↓ Urinary crystal deposit formation	[135]
	In vivo	EG-induced nephrolithiasis in rat	↓ Oxidative damage ↑ Serum paraoxonase 1 (PON1)	[119]

↑ demonstrates increasing trend; ↓ demonstrates deccreasing trend.
